# Time Pressure Increases Automation Reliance in a Face Matching Task

**DOI:** 10.1177/17470218251389943

**Published:** 2025-10-14

**Authors:** Alysha J. Hua, Peter J. B. Hancock, Daniel J. Carragher

**Affiliations:** 1 School of Psychology, Faculty of Health and Medical Sciences, The University of Adelaide, SA, Australia; 2 Psychology, Faculty of Natural Sciences, University of Stirling, Scotland, UK

**Keywords:** face identification, automated systems, human-algorithm teaming, decision-making

## Abstract

Automated facial recognition (AFR) systems are commonly used to help verify the identity of individuals, often in a one-to-one face matching task. The AFR system may be a decision aid, such that a human operator must ultimately approve the final identification decision. Previous research has shown that time pressure, a common operational factor in many applied settings, impairs human face matching accuracy. We investigated whether time pressure influences human reliance on AFR decisions in a face matching task, predicting that participants would show greater automation reliance as time pressure increased and the task became harder. Each participant (*n* = 129) completed a face matching task under blocks of high (2s), medium (5s), and low time pressure (10s), where the stimuli disappeared from the screen after the allocated time. For each pair of faces, participants made an initial identification decision without assistance. Participants were then shown an identification decision from a simulated AFR system (92.3% accurate), before they were asked to submit their final (assisted) decision to the same trial. As predicted, human accuracy improved with AFR assistance in all time pressure conditions, with the greatest improvements occurring under higher time pressure. But replicating previous studies, average aided human accuracy was below that of the AFR system alone. Moreover, assisted accuracy fell in all conditions when the AFR system provided an incorrect decision, suggesting participants struggled to correct system errors. Our results have implications for human oversight of AFR systems in applied face identification scenarios.

## Introduction

Accurately verifying the identity of an individual is important for many reasons including, but not limited to, maintaining national security at border control, identifying persons of interest in criminal investigations, protecting individuals from identity fraud, and preventing under-aged individuals from accessing age-restricted goods or premises. In many such settings, photographic identification documents (e.g., passports, driver’s licences, national identification cards) are used in the verification process. However, a common exploit is impersonation, where imposters obtain a genuine identity document that belongs to someone else, and based on the similarity of their appearance with the rightful owner, attempt to access goods and services as this other individual (National Crime Agency, 2015). Such identity fraud is often committed to facilitate further criminal offending. To mitigate the misuse of identity documents, employees at these institutions often perform one-to-one forensic face matching comparisons. This process asks the observer to determine whether the photo on the identity document is of the person presenting it for inspection. The outcomes are to conclude an identity ‘match’ (same person) or ‘mismatch’ (two different people). Though this forensic face matching task is common, decades of research have shown the task is surprisingly difficult when the faces are unfamiliar to the observer (e.g., Fysh & Bindemann, 2018a; Johnston & Bindemann, 2013; Kemp et al., 1997; Megreya & Burton, 2006).

Even under controlled and favourable laboratory conditions, the average human makes around 20% to 30% of errors in standard unfamiliar face matching tasks—that is, when the two faces being compared are not known to the participant (Burton et al., 2010; Kemp et al., 1997). Concerningly, the performance of professionals with face matching experience can be similar to individuals who have never completed a face matching task (Weatherford et al., 2021). While some highly trained professionals—facial examiners—reliably outperform novices (Phillips et al., 2018; White et al., 2015), even professionals with experience can miss a high ratio of fraudulent passports (White et al., 2014; Wirth & Carbon, 2017). Unfamiliar face matching accuracy can also be impacted by factors including variation in the view point of the faces (Bindemann et al., 2013; Estudillo & Bindemann, 2014), disguise or obstruction (Carragher & Hancock, 2020; Noyes & Jenkins, 2019), image quality (Strathie & McNeill, 2016), image colour (Bobak et al., 2019), the recency of the images (Megreya et al., 2013), and other information presented on the identification document (Feng & Burton, 2021; Trinh et al., 2022). These high error rates for the average human may help to explain the development of computer-based facial recognition algorithms (International Civil Aviation Organisation [ICAO], 2009).

Automated facial recognition (AFR) systems are computer algorithms that can compare the appearance of two face images to produce a value (often a similarity score), which is evaluated against a threshold to indicate whether the images likely show the same person or two different people (Mann & Smith, 2017; Noyes & Hill, 2021). Identity *verification*, the task pertinent to the current study, asks the algorithm to determine whether two submitted images belong to the same person, whereas *identification* functions typically see the algorithm compare a single probe image against an entire database of (often) known individuals. On tests with high-quality imagery, many state-of-the-art AFR systems can perform with exceptional accuracy (Grother et al., 2025), far exceeding the average human (Carragher & Hancock, 2023; O’Toole et al., 2007), and at a level similar to expert face matchers (Phillips & O’Toole, 2014; White et al., 2015). In addition to their typically impressive accuracy on these tasks, computers do not suffer many of the performance limitations that affect humans, such as performance deterioration as time spent on task increases (Alenezi & Bindemann, 2013; Alenezi et al., 2015; Fysh & Bindemann, 2017) nor do they experience concerns like sleep deprivation (Beattie et al., 2016). Furthermore, while humans demonstrate impaired face matching performance as time pressure increases (Bindemann et al., 2016; Fysh & Bindemann, 2017), the same time pressures are not relevant to these computer systems, which can return highly accurate outputs in fractions of a second. Thus, AFR systems can be useful in settings where prolonged, time-sensitive, identity screening is required, such as international airports, where such systems have been installed in passport ‘E-Gates’ (Fysh & Bindemann, 2018b).

Despite their often impressive accuracy, AFR systems make errors (Grother et al., 2025; Phillips et al., 2011), some of which can have life changing consequences for the affected individual (e.g., Hill, 2023). In an attempt to mitigate the possibility of unchecked algorithm errors, systems are often implemented such that a human provides oversight over the decisions made. In this configuration, the human and the AFR system are said to be a ‘human-algorithm team’ (Howard et al., 2020), though the system can be seen as an automated decision aid for the human observer, where they can choose to endorse or overturn the advice of the AFR system, but are responsible for the final identification decision made. Though these teams are already in operation, little is known in the scientific literature about the performance of human-algorithm teams on one-to-one face matching tasks (e.g., Barragan et al., 2022; Carragher & Hancock, 2023; Fysh & Bindemann, 2018b; Howard et al., 2020; O’Toole et al., 2007).

### Human-Algorithm Teaming in Face Matching

One early paper to explore the performance of human-algorithm teams in face matching reported that human decisions were biased toward the onscreen identification labels provided by a fictitious algorithm (Fysh & Bindemann, 2018b). Here, the algorithm gave the correct answer to 60% of trials, while 20% of decisions were incorrect, and 20% were ‘unresolved’. The authors found that human accuracy was higher on trials that were presented with the correct decision label, and lower when the label was incorrect, indicating that participants followed the onscreen labels, even when they were directed to ignore them. These results were supported by Howard et al. (2020), who discovered that prior identification decisions from both computer and human partners influenced the identification judgements made by participants. When the fictitious partner said that the two faces were an identity match, participants were more likely to make a match decision, even if the partner’s decision was incorrect. Both studies provided preliminary evidence that the human operator might be biased to follow the identification decision of the algorithm in a human-algorithm team.

In their face matching experiments, Carragher and Hancock (2023) used the similarity values generated by a real AFR system to inform the identity labels that were shown for each face pair. The authors reported that while AFR assistance improved human accuracy compared to an unassisted baseline, aided human performance was significantly lower than that of the same AFR system alone. Not only did the majority of human participants fail to catch errors from the AFR system, but they also overturned correct system decisions to create new errors (see also Bartlett et al., 2024). Similarly, Mueller et al. (2024) found that even when participants were shown decisions from an AFR system that were 100% accurate, participants did not achieve aided performance of 100%, suggesting that the average participant had difficulty in evaluating algorithm outputs. Carragher et al. (2024) showed that there are individual differences in effective use of the AFR, with those participants who showed high levels of relative trust in automation (Lee & Moray, 1994) achieving better aided accuracy than those who reported lower trust. Interestingly, the authors also reported that trust in the AFR improved after participants experienced using the system as a decision aid, where almost 50% of participants who initially did not trust the system for assistance, reported afterwards that they believed the system did help them. These results were used to suggest that reliance on the AFR system might change depending on task circumstances and experience.

While the majority of these studies have investigated the performance of human algorithm teams on standard face matching tasks, Barragan et al. (2022) asked human participants to complete a face matching task with assistance from an AFR system for faces covered by surgical masks. Their results showed larger shifts in confidence scores toward the advice of the AFR system when face masks were present on the faces than when masks were absent (Barragan et al., 2022). Considering face masks significantly impair human face matching accuracy, such that they make the task harder (Carragher & Hancock, 2020; Noyes et al., 2021; Ritchie et al., 2024), this result begs the question of whether reliance on AFR systems might increase as task difficulty increases (Schwark et al., 2010). If automation reliance increases with task difficulty, other operational factors that make face matching harder for humans might also influence the way humans use AFR systems as decision aids. Time pressure, which can result from constraints on viewing or responding to stimuli, is one such factor that is known to impair human face matching performance (Fysh & Bindemann, 2017). To date, there have been no studies exploring the effect of time pressure on human reliance on the AFR system.

### Time Pressure

Understanding reliance on AFR systems under time pressure may be particularly relevant in settings like passport control, where target times for processing each passenger have been reported (within 40-70 seconds; Department of Home Affairs, 2024). Although no research has explored how time pressure affects human reliance on AFR systems, previous research has shown that time pressure often decreases human performance in face matching tasks (Bindemann et al., 2016; Fysh & Bindemann, 2017; Özbek & Bindemann, 2011; White et al., 2015; Wirth & Carbon, 2017), and in other decision-making tasks (Rice et al., 2008; Rieger & Manzey, 2020; Rieger & Manzey, 2022). Several studies have reported that without time pressure, the average participant submits their face matching decisions in 5 to 6 seconds (e.g., Estudillo & Bindemann, 2014; Papesh & Goldinger, 2014). When stimuli were displayed for just 0.2s, Özbek and Bindemann (2011) reported an accuracy of approximately 60%, compared to near 90% accuracy when they were displayed for 2s or no time limit. Similarly, White et al. (2015) reported deteriorating face matching performance when stimuli were displayed for 2s compared to 30s, for both novices and professional facial examiners. These findings were supported by Bindemann et al. (2016) who found accuracy declined significantly when participants were asked to respond within 2s compared to their performance in trial blocks with 8s and 10s response targets. Finally, Fysh and Bindemann (2017) reported lower accuracy when participants were given 2s or 4s response time targets compared to their performance in blocks with 10s response targets. Regardless of their different methodological approaches to creating time pressure (i.e., showing stimuli for a fixed amount of time, or requiring responses within a specific period), these studies have all consistently shown that time pressure impairs human accuracy in unfamiliar face-matching tasks.

Additionally, time pressure has been found to decrease human performance in other decision-making tasks and increase reliance on automated decision aids. For example, one study found that time pressure decreased human performance in a medical visual search task, but that this negative effect was mitigated by using a highly reliable automated decision aid (Rieger & Manzey, 2022). The authors also report that participants searched the medical X-rays less when under higher time pressure and receiving assistance from an automated decision aid, thus indicating increased reliance on the system. Other literature has also reported a negative impact of time pressure, but in luggage screening tasks (Rieger & Manzey, 2020), with higher levels of automation reliance under higher time pressure (Rice et al., 2008). Finally, several authors have begun discussing the importance of human-automation teaming in time-constrained medical settings (Jacobs et al., 2021), and report that clinicians who use machine learning based systems view the automation as a partner rather than alternative to their own clinical judgement (Henry et al., 2022). Together, these studies emphasise the broad applicability of human–computer teaming paradigms to different decision-making contexts, and show how the factor of time pressure can influence reliance on automated decision aids.

### Research Aims

Our study aims to address this gap in the literature by investigating whether time pressure affects human use of AFR systems in a face-matching task. Previous research has shown that human face matching accuracy deteriorates as stimulus presentation time decreases (Fysh & Bindemann, 2017). Research has also reported that participants show greater reliance on automated aids under time pressure (Rice et al., 2008; Rieger & Manzey, 2020; Rieger & Manzey, 2022) or as task difficulty increases (Barragan et al., 2022; Schwark et al., 2010). H1: Therefore, we expect to find a significant interaction between time pressure (high, medium, low) and decision type (initial, final), such that assistance from the AFR system will lead to greater improvements in accuracy under higher time pressure. H2: However, as a result of the hypothesised increase in reliance, we also expect that participants will be most likely to endorse incorrect decisions made by the AFR system in the high pressure condition, followed by the medium condition, and then the low pressure condition. H3: Finally, we expect that the aided performance of participants in all three time pressure conditions will fail to reach that of the AFR system alone (92.3%), replicating previous research (Carragher & Hancock, 2023; Carragher et al., 2024; Mueller et al., 2024).

## Method

### Data Availability

All key components of this experiment were preregistered prior to data collection (https://osf.io/74mj3/). The data from this project are also available through the same link.

For transparency, we preregistered an initial experiment with the same aim, but in which the participants completed the short Glasgow Face Matching Test 2 (White et al., 2022). Though the pattern of the results matched our hypotheses, the effects were not statistically significant, despite a large sample size. We assessed that this non-significant result may have been due to a ceiling effect caused by unexpectedly high initial (unassisted) response accuracy among our participants on the short GFMT2 (see also Popovic et al., 2024). We report the method and results of our initial experiment in the supplementary materials, where the pre-registration link is also available. After selecting a different face matching task to avoid a ceiling effect, we sought to re-examine our hypotheses in the current study.

### Participants

Individuals were recruited through the online research platform *Prolific* (https://www.prolific.com/) to participate in the experiment in exchange for a small payment (Palan & Schitter, 2018). An *a priori* power analysis (G*Power; Faul et al., 2007) with recommended effect size specifications indicated that a total sample of 126 participants would be required to achieve 80% power to detect a medium sized interaction effect of η_p_^2^ = .06 with alpha set at .05. However, we slightly oversampled to allow for the application of our preregistered exclusion criteria and received consent from 145 unique individuals. Data were excluded from 6 participants who did not complete the entire experiment, 6 participants who accessed the experiment more than once, 1 participant who took more than 60 minutes to complete the task,^1^ and 3 participants who failed an attention check (see below). Therefore, the total sample included in our analyses consisted of 129 participants who were residents of the USA and reported fluency in English (85 female, 40 male, 2 non-binary, and 2 ‘other not listed’). Participants were aged between 18 and 73 years old (*M* = 39.7, *SD* = 12.6). This project received ethical approval from the Human Research Ethics Sub-Committee in the School of Psychology at the University of Adelaide (H-2023-01). On average, participants took 21.6 minutes (*SD* = 7.8) to complete the experiment.

### Design

The experiment had a within-participants design, such that all participants completed blocks of trials under high (2s), medium (5s), and low (10s) presentation time pressure conditions. Participants made an initial identification decision to each trial, before being shown the decision from the AFR system, and then submitting their final aided decision (see Hua & Carragher, 2024). As such, Presentation Time Pressure was a within-participants factor with three levels (high, medium, and low), and Decision Type was a within-participants factor with two levels (initial and final).

### Materials

#### Face Matching Task

Participants completed a face matching task that combined the 40 trials from the short Kent Face Matching Task (KFMT; Fysh & Bindemann, 2018a) and the 40 unfamiliar face pairs from the Stirling Famous Face Matching Task (SFFMT; Carragher & Hancock, 2020). Based on the data provided in the original publications, we removed the match (mated) and mismatch (non-mated) trial from this combined stimulus set with the highest normed accuracy, leaving 78 pairs of faces. These remaining trials were split into three blocks of 26 trials of equivalent difficulty, which each had 13 match and 13 mismatch trials. Each trial block was counterbalanced across presentation time pressure conditions. The presentation order of the trials within each block was randomised. Participants made their identification responses along a 6-point scale, which incorporated an identification decision (same, different) and a level of confidence (‘definitely’, ‘probably’, and ‘guess’; as in Mueller et al., 2024).

#### Presentation Time Pressure

All participants completed a block of trials under high, medium, and low presentation time pressure. Presentation time pressure was enforced by displaying a face pair for the specified duration, after which time they disappeared from the screen. Participants then made their initial and final decisions, without the stimuli on screen.

#### Automated Facial Recognition System

We used a real Deep Convolutional Neural Network (DCNN: an unpublished research system previously used in Carragher and Hancock, 2020, 2023) to generate similarity values for each pair of faces in this face matching task (values 0.4 and above are classified as match). Although the DCNN correctly resolved 100% of the KFMT and SFFMT trials, we used these similarity values to select three match and three mismatch trials (one in each time pressure block) that the simulated AFR system in the experiment would resolve incorrectly. These errors were the trials with similarity scores closest to the decision threshold of the real DCNN and allowed us to measure participants’ ability to overrule incorrect AFR decisions. The similarity values for mismatch trials ranged from -.12 to .39 (*M* = .15, *SD* = .11), while values for match trials ranged from .42 to .84 (*M* = .67, *SD* = .10). For mismatch trials that were turned into AFR error trials, the similarity scores were .39, .35, and .28. For match trials that were turned into AFR error trials, the similarity scores were .42, .47, and .47. This procedure for creating the ‘simulated’ AFR system is consistent with our previous research (Carragher & Hancock, 2023; Carragher et al., 2024). Thus, the accuracy of the simulated AFR system in the experiment was 92.3%, such that it correctly resolved 72/78 trials across the entire task (making 1 match and 1 mismatch error in each time pressure block). Importantly, all participants were told directly in the instructions that the decisions provided by the simulated AFR system would be correct on 92.3% of trials.

#### Attention Checks

Each time pressure block included one additional trial that consisted of two different politicians who had clear appearance and demographic differences. Participants did not see a decision from the AFR system on these trials, requiring that they made their identification response alone. Data from participants who made an incorrect ‘same’ response (regardless of confidence) as their initial decision to any of these attention check trials were removed from analysis.

### Procedure

Participants were told that they would be completing a face matching task with assistance from a simulated facial recognition system. In the instructions, participants were told truthfully that decisions from the simulated AFR system would be correct for 92.3% of trials. Participants were informed that the images in the face matching task would be ‘*shown for different amounts of time before they disappear off the screen’*, and that ‘s*ome pairs of faces will be presented for a short amount of time, while others will be presented for much longer’*. Though there were three blocks of different presentation time pressures, participants experienced the face matching task as one continuous run of trials. After the allocated presentation time, the face images disappeared from the screen, and participants provided their initial response to the question, ‘*Did these photos show the same person, or two different people’?* along the 6-point confidence scale. Without viewing the images again, participants were then shown their initial response along with the identification decision of the simulated AFR system (i.e., ‘*Facial Recognition System says: “same” or “different”’*), before submitting their final response to the trial.

### Analysis

#### Overall Accuracy

We calculated overall accuracy by determining whether the correct identification decision had been given to each trial, regardless of confidence. Overall accuracy is reported as a percentage, calculated by adding the correct match trial responses and correct mismatch trial responses, dividing the value by the total number of trials, then multiplying the result by 100. We calculated overall accuracy for each participant in each time pressure condition.

#### Confidence

Confidence was measured through participants selecting their identification response on a 6-point ‘same’ and ‘different’ scale. Identification responses of ‘same’ (definitely, probably, same) were given the values 1 to 3, while the values 4 to 6 were given to ‘different’ responses (guess, probably, definitely). Responses of ‘definitely’ were most confident, while ‘guess’ was the least confident response.

#### Area Under the Curve

Though our primary measure of performance is overall accuracy, calculating overall accuracy requires collapsing across the confidence information in our 6-point response scale. To supplement our analysis of accuracy, we also report area under the curve (AUC), a measure of sensitivity from signal detection theory that describes an individual’s ability to discriminate between match and mismatch trials at various decision thresholds (Macmillan & Creelman, 2005; Stanislaw & Todorov, 1999). An AUC value of 0.5 signals chance performance, while an AUC of 1.0 is perfect performance.

#### Decision Change

We report two measures of decision change. The first measure is identification decision change (as in Hua & Carragher, 2024). Identification decision change occurred when a participant’s identification decision changed between their initial and final decisions. Identification decision change was counted as a binary occurrence, such that decision change (changing from ‘same’ to ‘different’, or vice versa) either did or did not occur on a given trial.

The second measure is confidence change. Confidence change was measured using the entire 6-point response scale, and occurred whenever the participant gave a final decision that differed from their initial decision, regardless of whether the change resulted in a change of identification decision (i.e., an initial decision of ‘guess same’ and a final decision of ‘probably same’ is an example of confidence change).

## Results

### Planned Analyses

#### Time Pressure Influence on Overall Accuracy (H1)

**Figure 1.**
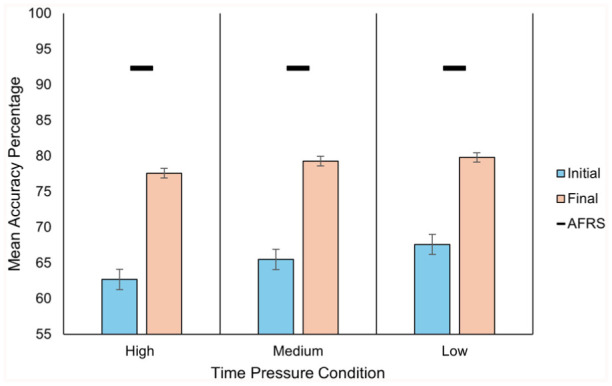
Average Accuracy Performance Across All Trials. *Note.* The error bars in this figure represent the standard error of the mean.

Overall accuracy in each time pressure condition is shown in Figure 1. To test our primary hypothesis that AFR system assistance would be most beneficial under greater time pressure, we performed a 3 (Time Pressure) x 2 (Decision Type) repeated measures ANOVA with overall accuracy as the dependant variable. The main effect of decision type was statistically significant, *F*(1, 128) = 228.11, *p* < .001, η_p_^2^ = .64. Final assisted decisions (*M* = 78.9%, *SD* = 8.7) were more accurate than initial decisions (*M* = 65.3%, *SD* = 8.7). The main effect of time pressure was also statistically significant, *F*(2, 256) = 8.77, *p* < .001, η_p_^2^ = .06, with greatest accuracy in the low time pressure condition (*M* = 73.7%, *SD* = 10.6), followed by the medium pressure (*M* = 72.4%, SD = *11*.1) and high time pressure conditions (*M* = 70.2%, *SD* = 10.6). Crucially, there was a significant interaction between decision type and time pressure, *F*(2, 256) = 5.07, *p* = .007, η_p_^2^ = .03. Bonferroni corrected post hoc comparisons showed that the improvement in the high time pressure condition was significantly larger than that in the low time pressure condition, *t*(129) = 3.18, *p_bonf_* = .005, *d* = 0.24. The magnitude of improvement did not differ between the high and medium, *t*(129) = 1.37, *p_bonf_* = .519, *d* = 0.10, or medium and low time pressure conditions, *t*(129) = 1.81, *p_bonf_* = .214, *d* = 0.14.

#### Endorsing AFR System Errors (H2)

**Figure 2.**
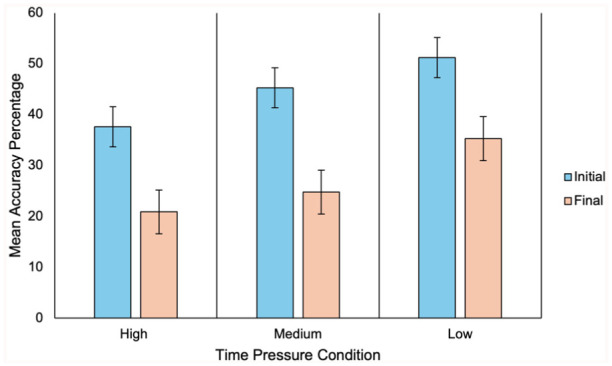
Average accuracy on trials answered incorrectly by the AFR system. *Note.* The error bars in this figure represent the standard error of the mean.

To test our second hypothesis that participants would be more likely to endorse AFR errors under greater time pressure, we performed a 3 x 2 ANOVA for mean accuracy on the trials answered incorrectly by the simulated AFR system (two per time pressure condition). There was a significant main effect of time pressure, *F*(2, 256) = 8.70, *p* < .001, η_p_^2^ = .06, such that accuracy on error trials decreased as time pressure increased. There was also a significant main effect of decision type, *F*(1, 128) = 119.76, *p* < .001, η_p_^2^ = .48 (see Figure 2); however, the pattern was reversed from our previous analysis (see Figure 1). That is, final aided accuracy was significantly *lower* than initial accuracy on error trials, which can be attributed to participants changing their correct initial decision to endorse the incorrect decision of the AFR system. However, the crucial interaction between time pressure and decision type was non-significant, *F*(1, 256) = 1.24, *p =* .290, η_p_^2^ = .01. Greater time pressure did not increase the number of AFR system errors accepted by participants, in contrast to our predictions.

#### Suboptimal Aided Performance (H3)

We compared the final accuracy achieved by participants in each time pressure condition with AFR system assistance to the level of performance achieved by the AFR system alone (92.3%) using one sample *t*-tests. As predicted, aided performance failed to reach the level of performance achieved by the AFR system alone in every time pressure condition: high (*M* = 77.6%, *SD* = 10.7), *t*(128) = −15.57, *p* < .001, *d* = −1.13, 95% CI [−1.61, −1.13], medium (*M* = 79.3%, *SD* = 11.0), *t*(128) = −13.41, *p* < .001, *d* = −1.18, 95% CI [−1.40, −0.95], and low (*M* = 79.8%, *SD* = 9.8), *t*(128) = −14.46, *p* < .001, *d* = −1.27, 95% CI [−1.50, −1.04]. These results replicate previous research (Carragher & Hancock, 2023; Carragher et al., 2024; Hua & Carragher, 2024).

### Exploratory Analyses

#### Time Pressure Influence—Area Under the Curve (H1)

**Figure 3.**
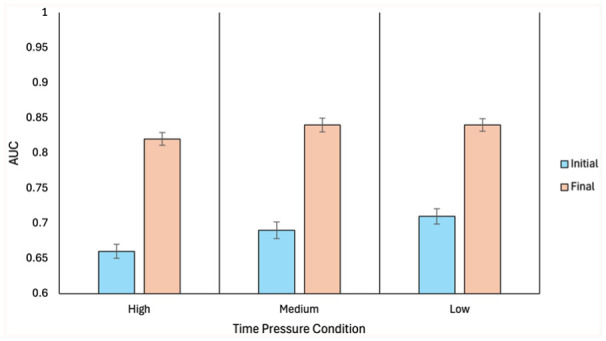
Average AUC Across All Trials. *Note*. The error bars in this figure represent standard error of the mean.

To support our analysis of accuracy above (H1), we performed an additional 3 (Time Pressure) x 2 (Decision Type) repeated measures ANOVA, but on AUC, a measure of sensitivity from signal detection theory (see Figure 3). The main effect of time pressure was statistically significant, *F(*1, 256) = 8.34, *p* < .001, η_p_^2^ = .06. The main effect of decision type was also statistically significant, *F(*1, 128) = 266.44, *p* < .001, η_p_^2^ = .68. Finally, there was a significant interaction between time pressure and decision type, *F(*1, 256) = 6.77, *p* = .001, η_p_^2^ = .05. These results confirm those reported above for overall accuracy. Participants benefited from AFR system assistance under all conditions, but the largest gains occurred under greater time pressure.

#### Identification Decision Change

We compared the number of identification decision changes that occurred in each time pressure condition using a one-way ANOVA. That is, how often participants changed the identification decision from their initial to their final judgment (i.e., changing from ‘same’ to ‘different’, or vice versa). The main effect of time pressure was significant, *F*(2, 256) = 5.69, *p* = .004, η_p_^2^ = .043. Participants changed their identification decision on 17.73% (*SD* = 3.39) of all trials in the high pressure condition, on 16.96% (*SD* = 3.26) of trials in the medium pressure condition, and on 14.79% (*SD* = 3.28) of trials in the low pressure condition. Bonferroni corrected post hoc comparisons confirmed that decision change occurred more frequently in the high pressure condition compared to low, *t*(129) = 3.20, *p_bonf_* = .005, *d* = 0.23, and in the medium pressure condition compared to low, *t*(129) = 2.51, *p_bonf_* = .040, *d* = 0.17, but there was no significant difference between the high and medium conditions, *t*(129) = 0.79, *p_bonf_* >.999, *d* = 0.06. Consistent with the gains in accuracy reported above, these results show that greater time pressure increased reliance on the decisions of the AFR system decision aid.

#### Confidence and Reliance

We first explored the confidence accuracy relationship by examining descriptive statistics of mean accuracy when each confidence level was selected. Descriptive statistics were examined rather than formal statistical analysis due to several participants never selecting a particular confidence level, resulting in missing data. The confidence data were relatively similar between time pressure conditions, so we present the data collapsed across each trial bock. A more in-depth exploration of confidence changes in each time pressure is available in the supplementary materials. Table 1 shows that accuracy was highest when participants selected a ‘definitely’ confidence response, compared to a ‘probably’ or ‘guess’ confidence response, which aligns with previous research (Stephens et al., 2017). The results show this pattern for both initial and final decisions, although final decisions show higher accuracy across all confidence levels.

**Table 1.** Frequency of Confidence Levels Selected.

	Decision Stage					
	Initial			Final		
Confidence	Accuracy	Count	Percentage	Accuracy	Count	Percentage
Guess	57.1%	2586	25.7	66.1%	2460	24.4
Probably	63.2%	4034	40.1	77.2%	3132	31.2
Definitely	73.9%	3442	34.2	87.1%	4470	44.4

*Note*. ‘Count’ refers to the frequency in which that confidence level was selected out of all trials (*n* = 10,062). ‘Percentage’ converts the count data into a percentage of total trials.

Next, we examined the change of confidence level from initial to final decisions in Table 1. We observe that participants were most likely to select a ‘probably’ level of confidence during initial decisions, followed by ‘definitely’ and ‘guess’, respectively. Conversely, confidence likely increased after viewing an AFR decision, demonstrated from participants being more likely to select a ‘definitely’ confidence response during final decisions, followed by ‘probably’ and ‘guess’, respectively.

Finally, we examined identification reliance on AFR system decisions in each confidence level in Table 2. That is, how often did participants change their identification decision to match that of the AFR system, for each initial confidence level. Data were split between trials where the AFR system presented a correct and incorrect identification decision, since relying on an incorrect system can lead to different consequences to relying on a correct system. We observed that participants were most likely change their identification decision to match the AFR when they were least confident. This pattern occurred in trials where a correct AFR decision was presented, and in trials where an AFR error was presented. These results suggest that lower confidence may lead to increased reliance on AFR. An important factor to note is what when reliance occurred in the AFR error trials, participants had initially made a correct identification decision. Thus, reliance led to decreased final accuracy and occurred more often when participants had lower confidence.

**Table 2.** Decision Change in Each Initial Confidence Level.

	Correct AFR Decision			AFR Error		
Confidence	Count	Change	Percentage	Count	Change	Percentage
Guess	2368	705	30	218	72	33
Probably	3703	594	16	331	51	15.4
Definitely	3217	214	6.7	225	15	6.7

*Note*. ‘Decision change’ refers to the frequency in which identification decision change occurred when participants selected their initial decision within that confidence level. ‘Percentage’ converts the count data into a percentage of occurrence from all trials where that confidence level was selected (i.e., when ‘guess’ was initially selected, reliance decision change occurred 777 times out of 2586 trials).

## Discussion

Our study explored the effects of time pressure on human performance in an automation-assisted face matching task. Time pressure significantly impaired unassisted human performance on the task, replicating previous findings (Bindemann et al., 2016; Fysh & Bindemann, 2017). As predicted, assistance from the AFR system significantly improved performance (both overall accuracy and AUC) in each time pressure condition. Though improvement was seen in all conditions, assistance from the AFR system was most beneficial when participants were under the greatest time pressure. Our analysis of decision change behaviours showed that participants were more likely to follow the decisions from the AFR system when they were under the greatest time pressure, which can explain this larger increase in performance. Interestingly, despite this greater reliance, participants were not more likely to incorrectly endorse AFR system errors under higher time pressure, but accuracy fell in all conditions when the AFR system presented an incorrect decision. However, this non-significant result may be attributed to the limited number of errors made by the simulated AFR system in this experiment (see below). Finally, it’s important to reiterate that the AFR-assisted accuracy of participants in every time pressure condition failed to reach the level of performance offered by the simulated AFR system alone, supporting our third hypothesis, and replicating previous findings (Carragher & Hancock, 2023; Carragher et al., 2024). Overall, these data suggest that an AFR system may be a useful decision aid in a time-pressured face matching task, but that the performance of the human-algorithm team is often sub-optimal (Bartlett et al., 2024).

Time pressure significantly impaired the accuracy of initial unassisted identification decisions, which is consistent with previous research (Bindemann et al., 2016; Fysh & Bindemann, 2017; White et al., 2015). Thus, although AFR-assistance was beneficial in every time pressure condition, it was more beneficial under high time pressure. However, final aided accuracy reached a similar level under all time pressure conditions, suggesting that time pressure may not have as large of an effect on decisions that can be assisted with automation. These results are similar to those reported by Rieger and Manzey (2022), who demonstrated decreased manual performance in a visual search task under time pressure, but found that automated decision support systems (DSS) mitigated these negative effects. Our results are also similar to a study that only found a negative impact of time pressure to performance in a manual luggage screening task, but not when assisted by an automated DSS (Rieger & Manzey, 2020). However, what these results show is that increased time pressure can lead to increased reliance on automated systems, which may be beneficial when the system is highly accurate, but not when the system has poor performance (e.g., Rice et al., 2008).

Since the simulated AFR system in the experiment was set to have an accuracy of 92.3%, the improvement in accuracy shows that participants often followed the correct decisions made by the system. Crucially, despite this improvement, AFR-assisted human performance failed to reach the same level of performance as the AFR system alone in every time pressure condition. This suboptimal aided performance in face matching tasks has been reported many times (Bartlett et al., 2024; Carragher & Hancock, 2023; Carragher et al., 2024; Hua & Carragher, 2024). This suboptimal performance has also been found in other areas of human-algorithm teaming research, where humans failed to reach the level of the automated DSS alone in a medical visual search task (Rieger & Manzey, 2022) and in a luggage screening task (Rice et al., 2008; Rieger & Manzey, 2020). Consequently, this result shows that participants did not simply follow every AFR decision. Rather, they must have overruled some of the system’s correct decisions. Notwithstanding this suboptimal aided performance, these results suggest that an AFR system decision aid might be particularly beneficial in scenarios where identification decisions are made under time pressure.

It is important, however, that greater reliance on the AFR system does not lead to increased acceptance of system errors. We had predicted that participants would be more likely to endorse errors made by the AFR system under higher time pressure due to their increased reliance on the system, but this prediction was not supported. However, there are three important points to consider along with this finding. First, aided accuracy fell in each time pressure condition when the AFR system made an error, demonstrating that most participants failed to overturn system errors. The lack of significant interaction in this analysis only shows that this failure of oversight was not any greater under high time pressure. Second, though the interaction was not statistically significant, we note that the pattern in Figure 2 is broadly consistent with our hypothesis. When the difference between initial and final accuracy is recalculated as a percentage decrease, we see that aided accuracy fell by 44.4% under high time pressure and 45.3% under medium time pressure, compared to 31.1% under low time pressure. Finally, we note that the AFR system only made two errors in each time pressure condition, which limited the opportunities for participants to potentially correct the system. This methodological choice was necessary to ensure the simulated AFR system had accuracy near that of modern systems, and remained a useful decision aid for participants to use. Future research using a face matching task with more trials (allowing more AFR system errors while maintaining high system accuracy) is needed to establish whether greater time pressure truly does not increase acceptance of AFR system errors, or whether this non-significant result is due to the limited number of error trials in this experiment.

Our exploratory analyses showed that participants changed their identification decision more often in the high time pressure condition than they did under medium or low time pressure. This result complements our analysis of performance and can be explained by the lower accuracy of initial decisions made under greater time pressure. Participants had more opportunity to engage in decision change under higher time pressure, simply because their initial decisions were less accurate, and therefore differed from those presented by the highly accurate AFR system. But these exploratory analyses also shed light on the way the AFR system output influenced confidence. Overall, participants were much more likely to shift their confidence towards the system than away from it, and were much more likely to increase confidence after the system agreed with their decision. However, given that lower accuracy was found in trials where participants had lower confidence, it is likely that participants found these trials more challenging. Similar results have been reported in previous literature, such as in Barragan et al. (2022) where participants often shifted confidence toward the system during a difficult identity verification task, or in Weger et al. (2015) who reported increased conformity to automated decisions when the task was more difficult. This exploratory analysis offers a detailed examination of decision change behaviours in an automation-assisted face matching task.

### Implications

While we found that participants could use the simulated AFR system to improve their own accuracy under time pressure, it is important to reiterate that the assisted performance of the average participant failed to reach the same level of accuracy as the simulated AFR system alone. This result must arise because they failed to correct the rare errors made by the AFR system, while also overturning correct decisions from the system. Although greater time pressure did not lead to increased acceptance of AFR system errors, human accuracy fell when the AFR system gave incorrect advice in each time pressure condition. Thus, our results have implications for the role of humans in forensic face matching tasks that incorporate algorithm assistance.

In some current implementations, the human operator is in place to provide oversight of decisions made by the AFR system. Our results in the current study, like those in previous studies, raise questions about the effectiveness with which humans can provide this necessary oversight over AFR systems in arrangements similar to this one. While this study, like others, offers a simplified laboratory version of human-algorithm teaming for face matching, which might not accurately represent a specific operational setting, the consistency with which sub-optimal aided performance is being reported across studies is notable—when interacting with simulated AFR systems, participants tend to limit the accuracy of the human-algorithm team (Carragher & Hancock, 2023; Carragher et al., 2024; Hua & Carragher, 2024; Mueller et al., 2024). This sub-optimal performance of the human-algorithm team is consistent with research in other fields such as medical decision making (Rieger & Manzey, 2022) or security luggage screening (Rice et al., 2008), and may also generalise to other settings. Previous research has shown that statistically fusing decisions that have been made independently by humans and algorithms can lead to significant gains in performance above what either agent achieves alone (O’Toole et al., 2007; Phillips et al., 2018). Further research is needed to find methods for achieving this elusive collaborative accuracy gain in interacting human-algorithm face matching teams.

### Limitations and Future Directions

Our decision to sample participants from the general population means that most, if not all, of these individuals likely had no professional face-matching experience or experience using AFR systems. Future research with participants who have face matching expertise or experience using automated systems is needed to assess the generalisability of these results. Yet, while some professionals—facial examiners—appear to have face matching abilities at the top end of the distribution (White et al., 2015), many studies have shown that experience performing face matching in a professional setting does not automatically lead to superior performance (Weatherford et al., 2021; White et al., 2014). As automated systems become increasingly common in applied settings, we must consider that people with little to no face-matching experience may be interacting with them. Though our testing of novice participants may be seen initially as a limitation, our results are valuable for understanding the implications of asking untrained individuals to provide oversight over such systems in future.

In the current study, we implemented time pressure as a limited opportunity to view the stimuli, but with an unlimited amount of time to make a response. While this approach is consistent with time pressure manipulations in previous studies (e.g., Özbek & Bindemann, 2011), it is not the only way time pressure has been conceptualised. Future research may apply the concept of global time pressure to our face matching task, in which participants are allocated a certain amount of time (e.g., 10 minutes) to complete all trials within the experiment, rather than enforcing a trial-by-trial time limit like the current study (e.g., 10 seconds per trial). This suggestion stems from prior research demonstrating that a global time pressure can be more detrimental than a strict trial-by-trial time limit (Wirth & Carbon, 2017). Moreover, global time pressures may be more applicable to many applied face matching or identity verification settings that enforce an overarching time pressure, rather than limiting the amount of time an operator can view the face. The results of such an experiment would show whether the particular type of time pressure in a setting may influence reliance on AFR systems.

## Conclusion

Accurate face matching is important for maintaining safety and security (Department of Home Affairs, 2023), and increasingly AFR systems are being introduced into applied settings (Noyes & Hill, 2021). Our results demonstrate that human face matching performance can deteriorate under time pressure, but that an AFR system can be a useful decision aid to improve human accuracy. The greatest improvement with AFR assistance was seen under the highest time pressures. However, there are important caveats to these favourable conclusions. Participants were likely to rely on the identification labels of the AFR system even when the system was incorrect, resulting in decreased accuracy. Furthermore, participants consistently failed to reach the accuracy of the AFR system alone, demonstrating that they were overruling correct decisions and failing to overrule errors. This sub-optimal aided performance occurred even though participants were truthfully told the accuracy of the simulated AFR system in the task instructions. The sub-optimal aided performance displayed in several studies now draws attention into exploring how humans can work with automation to increase the accuracy of the team, rather than inhibit the performance of the automation alone (e.g., Carragher & Hancock, 2023; Carragher et al., 2024). Consideration must be given to determining the role of humans in forensic face matching while working with AFR systems or performing oversight of automated decisions.

## Supplemental Material

sj-docx-1-qjp-10.1177_17470218251389943 – Supplemental material for Time Pressure Increases Automation Reliance in a Face Matching Task Supplemental material, sj-docx-1-qjp-10.1177_17470218251389943 for Time Pressure Increases Automation Reliance in a Face Matching Task by Alysha J. Hua, Peter J. B. Hancock and Daniel J. Carragher in Quarterly Journal of Experimental Psychology

## Notes

1. We deviated from our preregistration, which suggested an upper limit of 50 minutes (but was noted as being subject to revision). With this change, we retained three participants with completion times between 50 and 60 minutes.
