# Supplementary material for: Time Pressure Increases Automation Reliance in a Face Matching Task
Source: Q J Exp Psychol (Hove). 2025 Oct 14;79(7):1631–43. doi: 10.1177/17470218251389943 (PMC13310286; doi:10.1177/17470218251389943)
Supplement: sj-docx-1-qjp-10.1177_17470218251389943 – Supplemental material for Time Pressure Increases Automation Reliance in a Face Matching Task [file sj-docx-1-qjp-10.1177_17470218251389943.pdf]

Supplementary Materials*

Time Pressure Increases Automation Reliance in a Face Matching Task

*Alysha J. Hua[1], Peter J. B. Hancock[2], & Daniel J. Carragher[1]

[1]School of Psychology

Faculty of Health and Medical Sciences

The University of Adelaide

Adelaide, Australia

[2]Psychology

Faculty of Natural Sciences

University of Stirling

Scotland, United Kingdom

Total Word Count: 1,450 (approx.)

*Corresponding Author:
Alysha J. Hua
School of Psychology
Faculty of Health and Medical Sciences
University of Adelaide
Adelaide, South Australia, 5005
alysha.hua@adelaide.edu.au

Initial Experiment

As discussed in the main method, the experiment reported in the main text is a conceptual replication of an earlier experiment with an identical method, except for the face matching task. Although we found a pattern of results in this earlier experiment that were consistent with our hypotheses (the same as those in the main text) the interaction effect was not statistically significant, despite having a large sample size. When reviewing these results, we noted that the initial accuracy achieved by our participants without AFR-assistance was much higher than expected (White et al., 2022), which potentially lead to a ceiling effect that compressed the differences between conditions. This initial experiment was also pre-registered (https://osf.io/t8cfr), and we report the method and results here for transparency.

Method

Participants

We received consent from 97 unique individuals who were recruited online through the research participation system at our local institution. These individuals were first year undergraduate psychology students who participated in exchange for course credit. Individuals were ineligible to complete the study if they had participated in another face matching experiment run by our laboratory during the semester. Following our pre-registered criteria, we excluded data from 3 individuals who did not complete the experiment, 4 individuals who attempted the experiment more than once, 9 participants who took longer than 60 minutes to complete the task, and 2 participants who failed an attention check face matching trial. The final sample included in the analyses below consisted of 79 participants (M_{age} = 19.5, SD = 3.6, 58 female, 20 male, 1 non-binary), who took an average of 19.99 minutes (SD = 5.03) to complete the experiment.

Materials

This experiment was identical to that in the main text, with the only exception being that participants completed the short version of the Glasow Face Matching Task 2 (White et al., 2022). Please refer to the main text for all other methodological details.

The Glasgow Face Matching Task (GFMT2-S)

All participants completed the short version of the Glasow Face Matching Task 2 (GFMT2-S: White et al., 2022). The task consists of 80 trials in total, of which 40 are identity matches and 40 are identity mismatches. The normed data provided with the task report that average overall accuracy is 76.4%, with match accuracy (79%) slightly higher than mismatch accuracy (73.8%; White et al., 2022). However, we needed to create three blocks with an even number of trials in each. To achieve this requirement, we excluded the match and mismatch trial with the highest normed accuracy as per the data provided with the test (White et al., 2022), leaving a total of 78 trials. Each of our 3 blocks consisted of 26 trials (13 matches, 13 mismatches). Blocks were counterbalanced across time pressure conditions, such that each block was presented in all time pressure conditions across participants. As in the main experiment, the simulated Automated Facial Recognition (AFR) system made two errors in each block (one match trial, one mismatch trial), for an overall accuracy of 92.3%.

Results

Time Pressure (H1)

A 2 (decision phase: initial, final) x 3 (presentation time pressure: high, medium, low) repeated measures ANOVA on mean overall accuracy returned a significant main effect of decision type, $F(1,78) = 82.35$, $p < .001$, $\eta_p^2 = .514$, with initial decisions less accurate (M = 84.1%, SD = 3.7) than final decisions (M = 89.3%, SD = 3.1). The main effect of presentation time was also significant, $F(2,154) = 3.40$, $p = .037$, $\eta_p^2 = .042$, with accuracy lowest in the high pressure condition (M = 85.4%, SD = 3.5), followed by medium (M = 87%, SD = 3.3), and low (M = 87.8%, SD = 3.3). However, the interaction between decision type and

presentation time was non-significant, $F(2,154) = 0.38$, $p = .674$, $\eta_p^2 = .005$, suggesting the benefit of AFR system assistance was similar under each time pressure condition (see Figure 1).

Figure 1

Overall Face Matching Accuracy Across Time Pressure Conditions

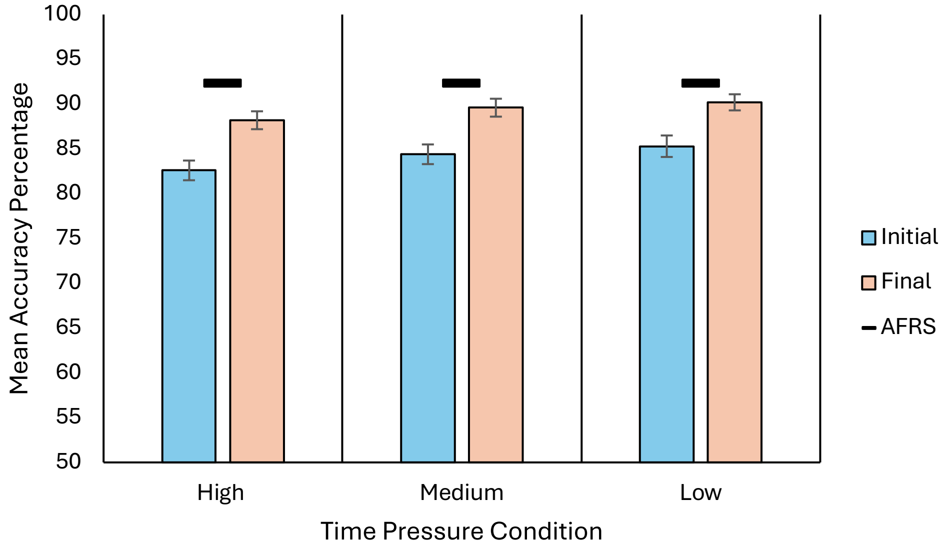

Note. Error bars represent standard error of the mean.

Endorsing AFR System Errors (H2)

Though we originally planned to examine descriptive statistics, we performed a 3 x 2 repeated measures ANOVA on trials answered incorrectly by the AFR. The main effect of decision type was statistically significant, $F(1,78) = 49.85$, $p < .001$, $\eta_p^2 = .390$. Contrary to the results above, the accuracy of final decisions was lower ($M = 66.2\%$, $SD = 4.7$) than initial decisions ($M = 81.6\%$, $SD = 3.88$), which can be explained by reliance on the incorrect system decision. The main effect of time pressure was non-significant, $F(2, 156) = 2.65$, $p = .074$, $\eta_p^2 = .033$, and there was no significant interaction between decision type and time pressure, $F(2,156) = 0.70$, $p = .500$, $\eta_p^2 = .009$ (see Figure 2).

Figure 2

Accuracy on AFR Error Trials

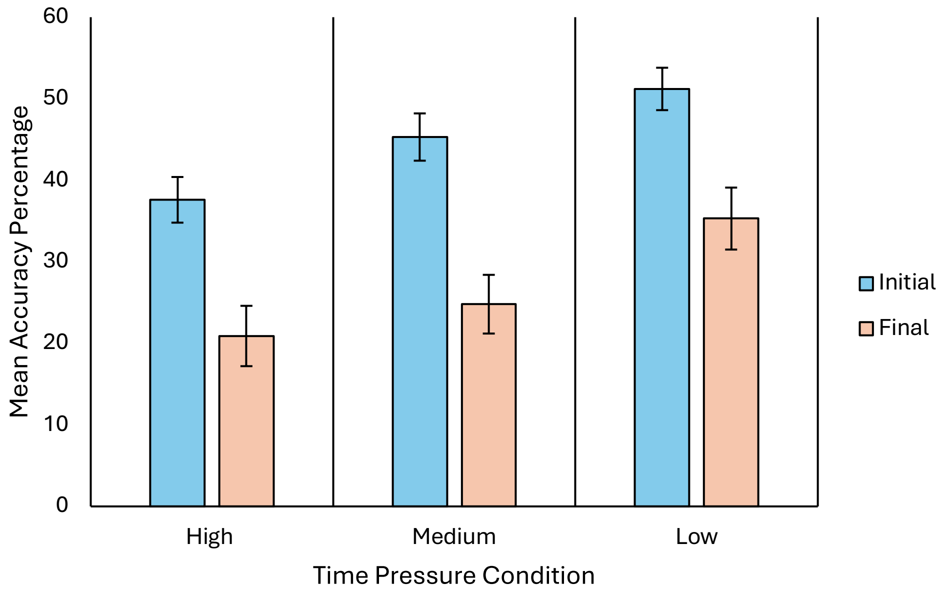

Note. Error bars represent standard error of the mean.

Confidence Change, Reliance, and Rejection (H3)

Subsequently, we explored whether confidence impacted reliance on AFR. We examine descriptive statistics across all time pressure conditions, because some participants did not use each confidence category in each presentation time condition during their initial responses, meaning there were missing data. It is important to note that, when the AFR was correct, participants who selected "definitely" were able to move toward the AFR only when their initial decision was incorrect. Conversely, when the AFR was incorrect, participants who selected "definitely" were able to move toward the AFR only when their initial decision was correct.

As expected, participants shifted their confidence toward the AFR system the most in trials where they were least confident (see Table 1). Shifts in confidence were more likely to

move toward the system than away from it, regardless of initial confidence or the accuracy of the AFR decision. Furthermore, participants were more likely to change their confidence in trials where the AFR displayed an incorrect identification decision.

Table 1

Confidence Change Depending on Initial Confidence

Confidence Change	Initial Confidence Category		
	Definitely	Probably	Guess
Correct AFRS Decision			
No Change	96.43%	61.70%	51.47%
Toward AFRS	2.46%	35.28%	47.64%
Against AFRS	1.11%	3.02%	0.89%
Total Trials	42.85%	35.53%	21.62%
Incorrect AFRS Decision			
No Change	67.37%	51.19%	49.14%
Toward AFRS	31.63%	44.64%	49.14%
Against AFRS	0%	4.17%	1.72%
Total Trials	40.09%	35.44%	24.47%

Note. The numbers displayed in this table demonstrate the percentage of trials within an initial confidence category where confidence change occurred.

Suboptimal Aided Performance (H4)

Using one sample *t*-tests, we compared the final accuracy in each time pressure condition with the level of performance achieved by the AFR system alone (92.3%) to test our fourth hypothesis. As predicted, participants failed to reach the performance of the system

in all time pressure conditions: high, $t(78) = -4.12$, $p < .001$, $d = .46$, 95%CI[-.69, -.23], medium, $t(78) = -2.76$, $p = .007$, $d = .31$, 95%CI[-.54, -.08], and low, $t(78) = -2.29$, $p = .025$, $d = .26$, 95%CI[-.48, -.03].

Main Text: Additional Results

Exploratory Analysis

Confidence Change and Reliance on AFR

To explore confidence shifts towards the AFR system under time pressure, we assessed the frequency of decision change toward (To) and away from the AFR system by each initial confidence category (i.e., definitely, probably, guess). We report confidence change during correct AFR system trials (top half of each table) separately to confidence change during AFR-error trials (bottom half of each table), since human operators are aiming to accept correct system decisions and overturn incorrect system decisions. Additionally, we report these findings based on whether the participant's initial identification response was incorrect (Table 2) or correct (Table 3). This split is necessary because initial responses limit whether participants can shift toward or away from the system when a 'definitely' confidence response was chosen (e.g., a participant who is incorrect with 'definitely' confidence cannot move away the system when it is correct, since they already disagreed maximally).

When looking at the results reported in Tables 2 and 3, we can see a number of patterns. First, participants were much more likely to shift their decision toward the AFR system rather than away, regardless of whether the AFR system presented a correct or incorrect decision, and regardless of whether their initial decision was correct. Second, participants were more likely to shift their decision toward that of the AFR system when their initial confidence was lower. Third, there were relatively few differences in decision change behaviours between the time pressure conditions. Of those that emerged, participants seemed more likely to change toward the AFR under high time pressure, when they gave a "probably" decision that was incorrect, but the AFR was correct (see Table 2).

Interestingly, there were cases in which the AFR system made an identification decision that was consistent with a participant's correct initial decision, but the participants

nonetheless shifted their final decision confidence away from the system. This pattern of responding occurred between 0-4% of trials, and was more likely to occur on trials where participants were initially correct than incorrect. These results suggest that participants may have doubted the accuracy of the presented AFR decision.

Table 2

Confidence Change when Participant Initial Decision was Incorrect

AFR Acc	Time Pressure	Initial Confidence								
		Definitely			Probably			Guess		
		Stay	To	Away	Stay	To	Away	Stay	To	Away
Correct 3065 33%	High	113 43.1%	149 56.9%	-	103 22.9%	345 76.9%	1 0.2%	107 28.2%	260 68.6%	12 3.7%
	Med	101 41.7%	141 58.3%	-	125 28.6%	308 70.5%	4 0.9%	87 25.7%	247 73.1%	4 1.9%
	Low	111 42.4%	151 57.6%	-	120 29.1%	288 69.9%	4 1%	84 29.4%	198 69.2%	4 1.4%
Error 428 55.3%	High	49 94.2%	-	3 5.8%	44 60.3%	29 39.7%	0	17 48.5%	18 51.4%	0
	Med	39 100%	-	0	36 52.2%	32 46.4%	1 1.4%	16 36.4%	28 63.6%	0
	Low	42 100%	-	0	26 45.6%	30 52.6%	1 1.8%	12 44.4%	15 55.6%	0

Note. First value presented is the count of total trials where confidence change occurred. Second value presented is the percentage of trials within the time pressure condition where confidence change occurred.

Table 3

Confidence Change when Participant Initial Decision was Correct

AFR Acc	Time	Confidence Category								
		Definitely			Probably			Guess		
		Stay	To	Away	Stay	To	Away	Stay	To	Away
Correct 6223 67%	High	726 98.8%	-	9 1.2%	375 48.8%	363 47.3%	30 3.9%	236 47.1%	264 52.7%	1 0.2%
	Med	784 97.5%	-	20 2.5%	440 52.2%	380 45.1%	23 2.7%	189 43.5%	244 56.2%	1 0.3%
	Low	893 98.1%	-	17 1.9%	407 51.1%	366 46%	23 2.9%	163 37.9%	267 62.1%	0
Error 346 44.7%	High	13 52%	12 48%	-	16 38.1%	26 61.9%	0	8 26.7%	21 70%	1 3.3%
	Med	16 57.1%	12 42.9%	-	17 37.8%	28 62.2%	0	15 34.1%	29 65.9%	0
	Low	20 51.3%	19 48.7%	-	24 43.6%	31 56.4%	0	15 39.5%	22 57.9%	1 2.6%

Note. First value presented is the count of total trials where confidence change occurred. Second value presented is the percentage of trials within the time pressure condition where confidence change occurred.
